# Differential Regulation of Proton-Sensitive Ion Channels by Phospholipids: A Comparative Study between ASICs and TRPV1

**DOI:** 10.1371/journal.pone.0122014

**Published:** 2015-03-17

**Authors:** Hae-Jin Kweon, Soo-Young Yu, Dong-Il Kim, Byung-Chang Suh

**Affiliations:** Department of Brain Science, Daegu Gyeongbuk Institute of Science and Technology (DGIST), Daegu, Republic of Korea; University of Hull, UNITED KINGDOM

## Abstract

Protons are released in pain-generating pathological conditions such as inflammation, ischemic stroke, infection, and cancer. During normal synaptic activities, protons are thought to play a role in neurotransmission processes. Acid-sensing ion channels (ASICs) are typical proton sensors in the central nervous system (CNS) and the peripheral nervous system (PNS). In addition to ASICs, capsaicin- and heat-activated transient receptor potential vanilloid 1 (TRPV1) channels can also mediate proton-mediated pain signaling. In spite of their importance in perception of pH fluctuations, the regulatory mechanisms of these proton-sensitive ion channels still need to be further investigated. Here, we compared regulation of ASICs and TRPV1 by membrane phosphoinositides, which are general cofactors of many receptors and ion channels. We observed that ASICs do not require membrane phosphatidylinositol 4-phosphate (PI(4)P) or phosphatidylinositol 4,5-bisphosphate (PI(4,5)P_2_) for their function. However, TRPV1 currents were inhibited by simultaneous breakdown of PI(4)P and PI(4,5)P_2_. By using a novel chimeric protein, CF-PTEN, that can specifically dephosphorylate at the D3 position of phosphatidylinositol 3,4,5-trisphosphate (PI(3,4,5)P_3_), we also observed that neither ASICs nor TRPV1 activities were altered by depletion of PI(3,4,5)P_3_ in intact cells. Finally, we compared the effects of arachidonic acid (AA) on two proton-sensitive ion channels. We observed that AA potentiates the currents of both ASICs and TRPV1, but that they have different recovery aspects. In conclusion, ASICs and TRPV1 have different sensitivities toward membrane phospholipids, such as PI(4)P, PI(4,5)P_2_, and AA, although they have common roles as proton sensors. Further investigation about the complementary roles and respective contributions of ASICs and TRPV1 in proton-mediated signaling is necessary.

## Introduction

Although the physiological pH level in the body is controlled by homeostatic mechanisms, transient and localized pH changes can occur in pain-generating pathological conditions and during normal synaptic activities [1,2]. Tissue acidosis is triggered during pain-producing inflammation, infection, ischemic stroke, and tumor development [1,3]. Severe ischemic stroke can reduce the extracellular pH level to 6.3 or even lower [4]. Since local pH changes occur during normal brain activities [5], detection of pH fluctuations in the brain is also important. The proton has been recently reported as a neurotransmitter that can induce excitatory postsynaptic currents and regulate synaptic plasticity in the lateral amygdala [2]. For that reason, it is not surprising that intrinsic systems capable of sensing pH fluctuations exist in our bodies.

Acid-sensing ion channels (ASICs), which belong to the epithelial Na^+^ channel (ENaC)/degenerin (DEG) superfamily of ion channels, are responsible for perception of pH changes in the central nervous system (CNS) and the peripheral nervous system (PNS) [1]. These channels are voltage-independent, proton-gated cation channels mainly permeable to Na^+^ ion. Until recently, it has been reported that four genes, ACCN2, ACCN1, ACCN3, and ACCN4, encode at least six subunits, ASIC1a, ASIC1b, ASIC2a, ASIC2b, ASIC3, and ASIC4 [1,6]. Each subunit consists of two transmembrane (TM) domains, a large extracellular loop, and short intracellular N- and C-termini. They can form homo- or hetero-trimeric channels, while ASIC2b and ASIC4 only contribute to the functional properties of heteromeric channels containing each of them rather than the formation of homomeric channels. Capsaicin- or thermal stimuli-activated transient receptor potential vanilloid 1 (TRPV1) channels are also proton sensors primarily expressed in sensory neurons [7–9]. These polymodal signal integrators have received attention as therapeutic targets for pain. Four subunits are required to form a functional TRPV1 channel. Each subunit is composed of six TM domains, a pore domain between TM5 and TM6, and intracellular N- and C-termini [9]. When opened by thermal or chemical noxious stimuli, these channels are permeable to nonselective cations with high Ca^2+^ permeability. Ca^2+^ influx through TRPV1 induces robust depletion of phosphatidylinositol 4-phosphate (PI(4)P) and phosphatidylinositol 4,5-bisphosphate (PI(4,5)P_2_) via the activation of phospholipase C-δ (PLCδ) isoforms and leads to desensitization of channels [10,11]. TRPV1 has putative proton binding sites on the extracellular face of the channel protein [7,9] as ASICs [12]. TRPV1-mediated proton sensing is physiologically relevant to perception of nociceptive and inflammatory pain signaling in primary afferent neurons [13].

A signaling lipid, PI(4,5)P_2_, which is a minor acidic phospholipid in the inner leaflet of the eukaryotic cellular membranes, has received attention as a functional cofactor for membrane receptors and ion channels [14,15]. Cleavage of PI(4,5)P_2_ by receptor-activated PLC generates two second messengers: membrane-bound lipid diacylglycerol (DAG) and soluble inositol 1,4,5-trisphosphate (IP_3_). Depletion of PI(4,5)P_2_ during PLC signaling also inhibits the currents of several ion channels [14], including inwardly rectifying K^+^ (Kir) channel [16], KCNQ channel [17,18], voltage-gated Ca^2+^ channel (VGCC) [19], ENaC [20–22], and several members of the TRP channel family [23].

Although many interesting studies revealed that the plasma membrane (PM) phosphoinositide PI(4,5)P_2_ is a regulator of TRPV1 channels, whether PI(4,5)P_2_ has inhibitory or potentiating effects on their activities has been debated over the past decade [10,11,24–30]. In the case of ASICs, whether these channels have dependence on the phosphoinositides for their function has not been determined yet [1]. Dorofeeva et al. (2009) [31] reported that homomeric ASIC1a channels can be inhibited by the activation of G_q_-coupled M_1_ muscarinic receptor (M_1_R), which leads to hydrolysis of both PI(4)P and PI(4,5)P_2_ through the activation of PLCβ enzymes [32,33]. However, Li et al. (2012) [34] were not able to observe the decrease of ASIC1a currents during muscarinic receptor activation. Therefore, it is necessary to further examine the dependence of ASICs on membrane phospholipids for their function.

In this study, we focused on determining the sensitivities of ASICs toward phospholipids by comparing them to proton-sensitive TRPV1 channels. We used the recently developed translocatable pseudojanin (PJ) system [35] for investigating the sensitivities of ASICs to PM PI(4)P and PI(4,5)P_2_. In addition, we generated a novel inducible 3-phosphatase that can specifically dephosphorylate PM phosphatidylinositol 3,4,5-trisphosphate (PI(3,4,5)P_3_) and investigated PI(3,4,5)P_3_ sensitivities of ASICs and TRPV1 channels in intact cells. Our studies demonstrate that the sensitivities of proton-sensitive ion channels toward PM phospholipids differ significantly depending on the type of channels.

## Materials and Methods

### Plasmids

Mouse cDNA clones of ASIC1a, ASIC2a, and ASIC3 were generously given to us by Michael J. Welsh (University of Iowa, Iowa city, Iowa). For the C-terminal fusion of GFP to each ASIC subunit, the cDNAs encoding ASIC1a, ASIC2a, and ASIC3 were amplified by the PCR using the primers (forward primer, 5’-GAATTCATGGAACTGAAGACCGAG-3’, reverse primer, 5’-GGATCCCGGCAGGTAAAGTCCTCAAA-3’, for ASIC1a-GFP; forward primer, 5’-GAATTCATGGACCTCAAGGAGAGC-3’, reverse primer, 5’-GGATCCCGGCAGGCAATCTCCTCCAG-3’, for ASIC2a-GFP; and forward primer, 5’-GAATTCATGAAACCTCCCTCAGGA-3’, reverse primer, 5’-GGTACCGTGAGCCTTGTCACGAGGTA-3’, for ASIC3-GFP), and inserted into the pEGFP-N1 (Clontech) vector. For the generation of CF-PTEN chimera, the DNA fragment encoding codons 22–403 was amplified by the PCR from Ci-VSPTEN21 [36], a kind gift from Carlos A. Villalba-Galea (Virginia Commonwealth University, Richmond, Virginia), using forward primer, 5’-AAGCTTCGGACTTAGACTTGACCTATA-3’, reverse primer, 5’-GGATCCGACTTTTGTAATTTGTGAA-3’, and fused to the C-terminus of CFP-FKBP. The following plasmids were generously given to us: Pseudojanin (PJ), PJ-Dead, PJ-Sac, INPP5E, LDR, PLCδ1-PH-GFP (from Bertil Hille, University of Washington School of Medicine, Seattle, Washington); Osh1-PH-GFP (from Deok-Jin Jang, Kyungpook National University, Sangju, Korea); rat TRPV1 with internal ribosome entry site EGFP and rat TRPV1 without GFP (from Jae-Yong Park, Korea University, Seoul, Korea); and Btk-PH-GFP (from Carlos A. Villalba-Galea, Virginia Commonwealth University, Richmond, Virginia).

### Cell Culture and Materials

TsA201 cells, derived from human embryonic kidney 293 cells (*293tsA1609neo*) by stably transfecting with the SV40 T-antigen [37] were obtained from Bertil Hille (University of Washington School of Medicine, Seattle, Washington). The cells were cultured in DMEM supplemented with 10% FBS and 0.2% penicillin/streptomycin at 37°C with 5% CO_2_ and transiently transfected using Lipofectamine 2000 (Invitrogen) with various cDNAs. For homomeric ASICs expression, cells were transfected with cDNA encoding ASIC1a-GFP, or ASIC2a-GFP, or ASIC3-GFP. For heteromeric ASICs expression, cells were transfected with different ASIC subunits in a 1:1 molar ratio, and 0.2 μg of cDNA encoding GFP was co-transfected as a marker for successfully transfected cells. GFP-positive cells were selected for recording the ASIC currents. For TRPV1 expression, cells were transfected with cDNA encoding TRPV1 with or without GFP. When needed, 0.2 μg of cDNA encoding tetrameric red fluorescence protein (DsRed) was co-transfected with TRPV1 as a marker for successfully transfected cells. The next day, cells were plated onto poly-L-lysine (0.1 mg/ml, Sigma) coated chips, and the fluorescent cells were studied within 2 days after transfection. Rapamycin (LC Laboratories), arachidonic acid (Sigma), amiloride (Tocris), and capsazepine (Sigma) were dissolved in DMSO (Sigma) to make stock solutions. Stock solutions were diluted in Ringer’s solution before use.

### Patch Clamp Recording

The whole-cell configuration of the patch clamp technique was used to voltage-clamp at room temperature (22–25°C). Electrodes pulled from glass micropipette capillaries (Sutter Instrument) had resistances of 2–2.5 MΩ, and series resistance errors were compensated by > 60%. Fast and slow capacitances were compensated before the application of test-pulse. Recordings were performed using a HEKA EPC-10 amplifier with pulse software (HEKA Elektronik). The external Ringer’s solution used for recording ASIC currents contained 160 mM NaCl, 5 mM KCl, 1 mM MgCl_2_, 2 mM CaCl_2_, and 10 mM HEPES, adjusted to pH 7.4 with tetramethylammonium hydroxide. For acidic solutions below pH 6.0, HEPES was replaced with MES. The pipette solution contained 140 mM KCl, 5 mM MgCl_2_, 10 mM HEPES, 0.1 mM 1,2-bis(2-aminophenoxy)ethane-*N*,*N*,*N’*,*N’*-tetraacetic acid (BAPTA), 3 mM Na_2_ATP, and 0.1 mM Na_3_GTP, adjusted to pH 7.4 with KOH. ASIC currents were recorded by holding the cell at -70 mV. The external Ringer’s solution used for recording TRPV1 currents contained 150 mM NaCl, 5 mM KCl, 1 mM MgCl_2_, 2 mM EGTA, 10 mM Glucose, and 10 mM HEPES, adjusted to pH 7.4 with NaOH. The pipette solution contained 135 mM CsCl, 5 mM MgCl_2_, 10 mM HEPES, 5 mM EGTA, 5 mM Na_2_ATP, and 10 mM Glucose, adjusted to pH 7.4 with CsOH. TRPV1 currents were recorded by holding the cell at -80 mV. The following reagents were obtained: BAPTA, Na_2_ATP, Na_3_GTP, EGTA, CsOH and tetramethylammonium hydroxide (Sigma), HEPES (Calbiochem), MES (Alfa Aesar), and other chemicals (Merck).

### Confocal Imaging

TsA201 cells were imaged 1–2 days after transfection on poly-L-lysine coated chips with a Carl Zeiss LSM 700 confocal microscope (Carl Zeiss AG) at room temperature. The external Ringer’s solution contained 160 mM NaCl, 2.5 mM KCl, 2 mM CaCl_2_, 1 mM MgCl_2_, 10 mM HEPES, and 8 mM Glucose, adjusted to pH 7.4 with NaOH. For time courses, cell images were scanned with a 40 X (water) objective lens at 512 X 512 pixels using digital zoom. During time course experiments, images were taken every 5 s. Quantitative analysis of the cytosolic fluorescence intensity was performed using the ‘measure’ tool for the region of interest in ZEN 2012 lite imaging software (Carl Zeiss MicroImaging). All confocal images were transferred from LSM5 to JPEG format, and raw data from time courses was processed with Microsoft Office Excel 2012 (Microsoft) and Igor Pro (WaveMetrics, Inc.).

### Data Analysis

Data acquisition and analysis used Pulse/Pulse Fit software in combination with an EPC-10 patch clamp amplifier (HEKA Elektronik). Further data processing was performed with Microsoft Office Excel 2012 (Microsoft) and Igor Pro (WaveMetrics, Inc.). All quantitative data are expressed as the mean ± SEM. Comparisons between two groups were analyzed using Student’s *t*-test. Comparisons among more than two groups were analyzed using one-way ANOVA followed by Bonferroni post-hoc test. Comparisons among more than two groups with two independent variables were analyzed using two-way ANOVA followed by Bonferroni post-hoc test. Differences were considered significant at the * *P* < 0.05, ** *P* < 0.01, and *** *P* < 0.001 levels, as appropriate.

## Results

### The Activities of TRPV1 Channels are Dependent on Membrane Phosphoinositides

**Fig 1:**
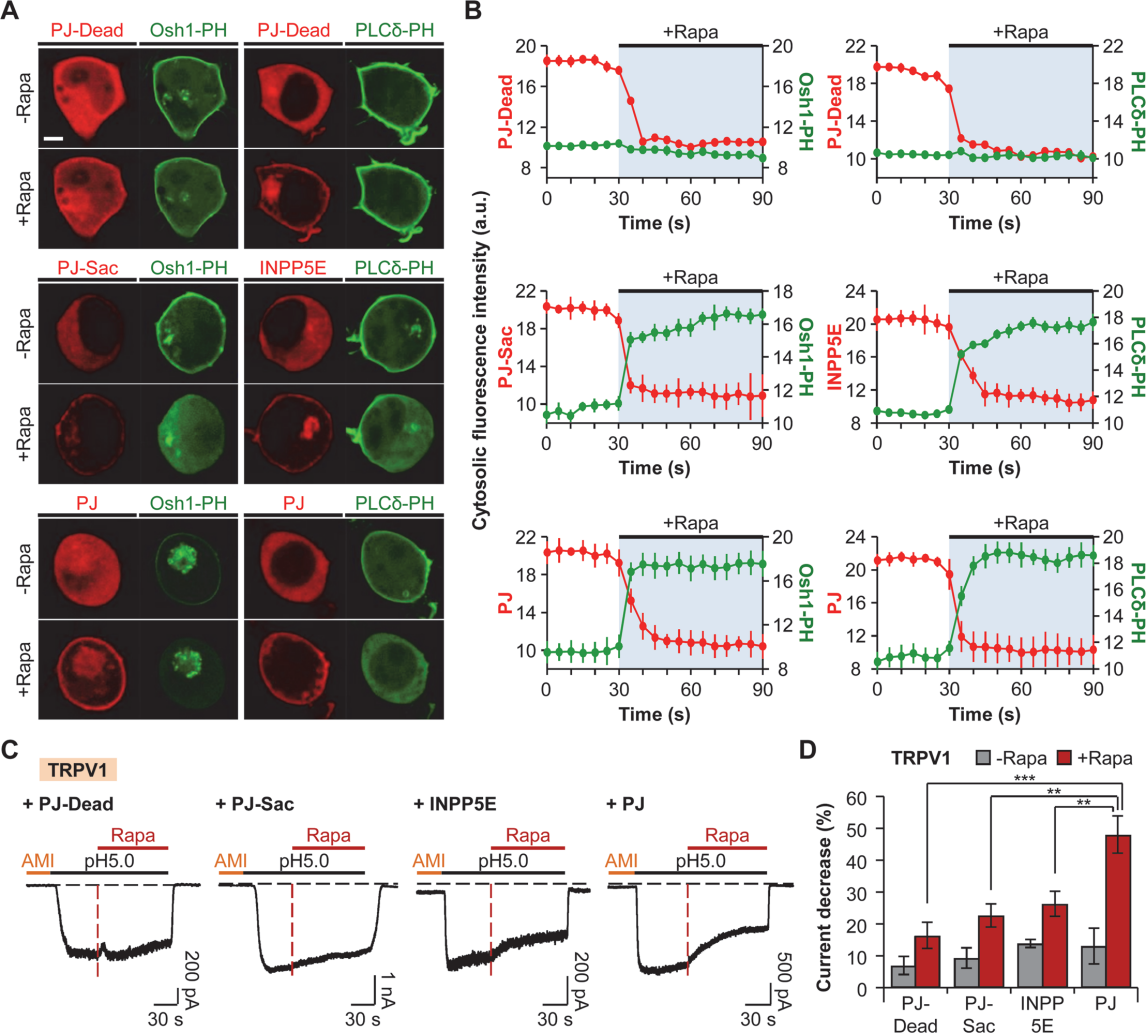
The activity of TRPV1 is dependent on phosphoinositides. (A) Confocal images of cells expressing PJ-Dead (*top*), PJ-Sac (*middle*), INPP5E (*middle*), or PJ (*bottom*) with LDR and respective biosensors for PI(4)P (Osh1-PH-GFP) or PI(4,5)P_2_ (PLCδ1-PH-GFP). Images before and after the addition of rapamycin (1 μM) for 60 s (Scale bar, 5 μm). (B) Cytosolic fluorescence intensities of RFP (*red*) and GFP (*green*) for the cells in (A). The values of the Y-axis use an arbitrary unit. Cells expressing PJ-Dead (*top*), PJ-Sac (*middle*), INPP5E (*middle*), or PJ (*bottom*) (n = 3, respectively). (C) TRPV1 currents triggered by prolonged extracellular pH drop to 5.0 for 150 s. Rapamycin (1 μM) was co-applied for 90 s during the acid stimuli. Amiloride (300 μM) was pretreated for 30 s before the pH pulse. Black dashed line indicates the zero current level. Red dashed line indicates the point of rapamycin application. (D) Percentage of current decrease in (C) during 45 s of acidification before (grey) and after (red) rapamycin addition (n = 12 for PJ-Dead; n = 14 for PJ-Sac; n = 12 for INPP5E; n = 10 for PJ). ** *P* < 0.01 and *** *P* < 0.001, with two-way ANOVA followed by Bonferroni post-hoc test and one-way ANOVA followed by Bonferroni post-hoc test. Data are mean ± SEM.

To assess the requirement of PM phosphoinositides for the activities of proton-sensitive ion channels, we employed the chemically-inducible dimerization (CID) system. Rapamycin-induced dimerization of FKBP (FK506 binding protein) and FRB (FKBP-rapamycin binding domain of mTOR) can be used to rapidly and irreversibly recruit the enzyme of interest to the target region inside the cell [38]. We applied the recently described fusion protein mRFP-FKBP-Pseudojanin (PJ), which contains both inositol polyphosphate-5-phosphatase E (INPP5E) and sac1 phosphatase, and thus, the recruitment of which to the PM leads to simultaneous depletion of PI(4,5)P_2_ and PI(4)P [35]. First, we tested the activity of the PJ construct by using the pleckstrin homology (PH) domain of PLCδ1 (PLCδ1-PH-GFP) and oxysterol-binding protein homologues (Osh1-PH-GFP) as indicators for PM PI(4,5)P_2_ and PI(4)P, respectively. PJ was localized to the cytoplasm when expressed in tsA201 cells. Recruiting PJ to the PM anchor LDR (N-terminal myristoylation and palmitoylation modification sequence of a Lyn kinase coupled to FRB domain) by the addition of 1 μM of rapamycin (Rapa) for 60 s resulted in the release of both Osh1-PH and PLCδ-PH to the cytoplasm in each separate confocal experiment (Fig. 1A, *bottom*). The cytosolic fluorescence intensity of PJ rapidly declined upon rapamycin addition, while that of Osh1-PH or PLCδ-PH increased (Fig. 1B, *bottom*). We also confirmed that the recruitment of PJ-Sac (INPP5E domain is inactivated by mutation to specifically dephosphorylate PI(4)P [35]) resulted in the release of Osh1-PH from the PM and increase in the cytosolic fluorescence intensity of Osh1-PH (Fig. 1A and B, *middle*). Recruitment of INPP5E to the PM decreased the PI(4,5)P_2_ level, which was confirmed by the translocation of PI(4,5)P_2_ probe to the cytoplasm with the increase in the cytosolic intensity of PLCδ-PH (Fig. 1A and B, *middle*). However, recruiting the cytosolic PJ-Dead, a chimera with inactivated sac1 and INPP5E [35], to the PM had no effects on the localization of Osh1-PH or PLCδ-PH (Fig. 1A, *top*). Consequently, the cytosolic fluorescence intensities of Osh1-PH and PLCδ-PH remained unchanged following the addition of rapamycin (Fig. 1B, *top*).

We applied this system on acid-evoked TRPV1 currents to test the regulatory aspects of phosphoinositides reported by previous studies [10,11,24–30]. Since tsA201 cells have been reported to have endogenous ASIC currents [39], the cells transiently expressing TRPV1 and respective PJ system constructs were preincubated by amiloride (AMI), a general inhibitor of ASICs, before the acid stimulation to selectively measure the TRPV1 currents. After the preincubation of cells with extracellular solution containing 300 μM of amiloride for 30 s, the cells were stimulated by pH 5.0 solution for 60 s; then, 1 μM of rapamycin was co-applied with pH 5.0 solution for 90 s. Since we observed variable desensitization among the cells during prolonged acidification before the addition of rapamycin, we first compared how much the currents decrease during acidification in the absence of rapamycin among four groups (PJ-Dead, PJ-Sac, INPP5E, and PJ). TRPV1 currents usually reached the maximum current amplitude within 15 s after the currents were activated; thus, we compared the current decrease during 45 s of acidification after reaching the maximum current amplitude. The currents of TRPV1 in cells expressing PJ-Dead or PJ-Sac were desensitized by 7 ± 3% (n = 12) or 9 ± 3% (n = 14), respectively (Fig. 1C-D). When the cells were transfected with INPP5E or PJ, the currents were decreased by 14 ± 1% (n = 12) or 13 ± 6% (n = 10), respectively (Fig. 1C-D). Desensitization rates of currents showed no statistically significant differences among the four groups before the application of rapamycin. The currents in cells expressing PJ-Dead, PJ-Sac, and INPP5E were decreased by 16 ± 4% (n = 12), 23 ± 4% (n = 14), and 26 ± 4% (n = 12), respectively, during 45 s of acidification right after rapamycin addition (Fig. 1C-D). However, translocation of PJ to the PM by the application of rapamycin decreased TRPV1 currents by 48 ± 6% (n = 10) during 45 s of acidification (Fig. 1C-D). These results suggest that the activities of TRPV1 channels are diminished by simultaneous breakdown of both PM PI(4)P and PI(4,5)P_2_, as previously reported [11,35].

### The Activities of ASICs are Independent from PI(4)P and PI(4,5)P_2_

**Fig 2:**
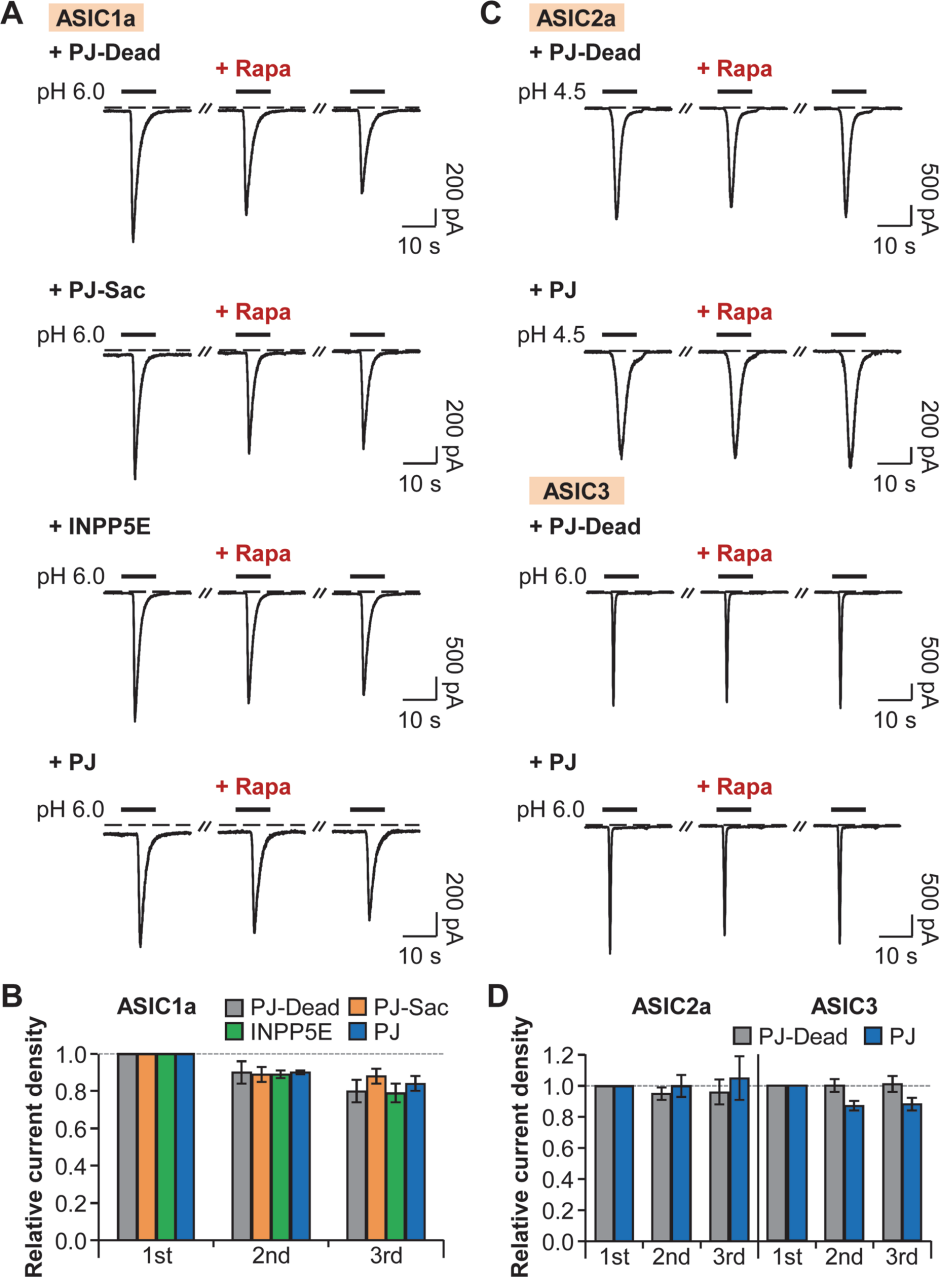
Homomeric ASIC currents are insensitive to PI(4)P and PI(4,5)P_2_. (A) ASIC1a currents evoked by repetitive rapid extracellular pH change from 7.4 to 6.0 for 10 s with time intervals of 120 s in cells expressing LDR and either PJ-Dead, PJ-Sac, INPP5E, or PJ. Rapamycin (1 μM) was bath-applied for 60 s, and then normal extracellular solution was perfused for 10 s right before the second pulse to minimize possible side effects of rapamycin. Dashed line indicates the zero current level. (B) Relative current density measured for the cells in (A) (n = 6, respectively). Current density of each pulse was divided by that of the first pulse. There is no statistical significance with two-way ANOVA followed by Bonferroni post-hoc test. (C) ASIC2a or ASIC3 current traces evoked by pH drop to 4.5 or 6.0 for 10 s. (D) Relative current density measured for the cells in (C) (n = 6, respectively). There is no statistical significance with two-way ANOVA followed by Bonferroni post-hoc test. Data are mean ± SEM.

Next, we examined whether ASICs also require phosphoinositides for their function as TRPV1 channels. ENaC belongs to the same superfamily of ion channels as ASICs and is known to be regulated by PM PI(4,5)P_2_ and PI(3,4,5)P_3_ [20–22]. That means, ASICs could have sensitivities toward phosphoinositides. To test this, we employed the PJ system and observed the activities of homomeric and heteromeric ASICs. The cells transiently expressing each GFP-tagged ASIC subunit (ASIC1a, or ASIC2a, or ASIC3) and respective PJ systems were repetitively activated by extracellular acidification from pH 7.4 to pH 6.0 (ASIC1a or ASIC3) or to pH 4.5 (ASIC2a) for 10 s with 2 min time intervals. As previously reported, GFP fusion to the C-terminus of ASIC subunit did not affect the electrophysiological properties of wild-type ASIC currents expressed in tsA201 cells [40]. For recruiting the interested enzyme to the PM, 1 μM of rapamycin was perfused for 60 s before the second pH pulse. To minimize possible side effects of rapamycin, normal extracellular solution was perfused right after the application of rapamycin for 10 s just before the second pH pulse. First, we confirmed that, as a control, the recruitment of PJ-Dead to the PM anchor does not affect the repetitive proton-activated ASIC1a currents except for tachyphylaxis (reduction in current amplitude with repeated stimulation), a unique property of homomeric ASIC1a channels [41] (Fig. 2A-B). Then, we observed that translocation of PJ-Sac or INPP5E to the PM to specifically deplete PM PI(4)P or PI(4,5)P_2_, respectively, had no effects on the relative current density of homomeric ASIC1a channels (Fig. 2A-B). Simultaneous depletion of both PI(4)P and PI(4,5)P_2_ by PJ also had no significant effect on the successively triggered ASIC1a currents (Fig. 2A-B). We found that neither ASIC2a nor ASIC3 homomeric channels were affected by the recruitment of PJ to the PM (Fig. 2C-D), allowing us to conclude that unlike proton-sensitive TRPV1 channels, the activities of homomeric ASICs are independent of PM PI(4)P and PI(4,5)P_2_.

**Fig 3:**
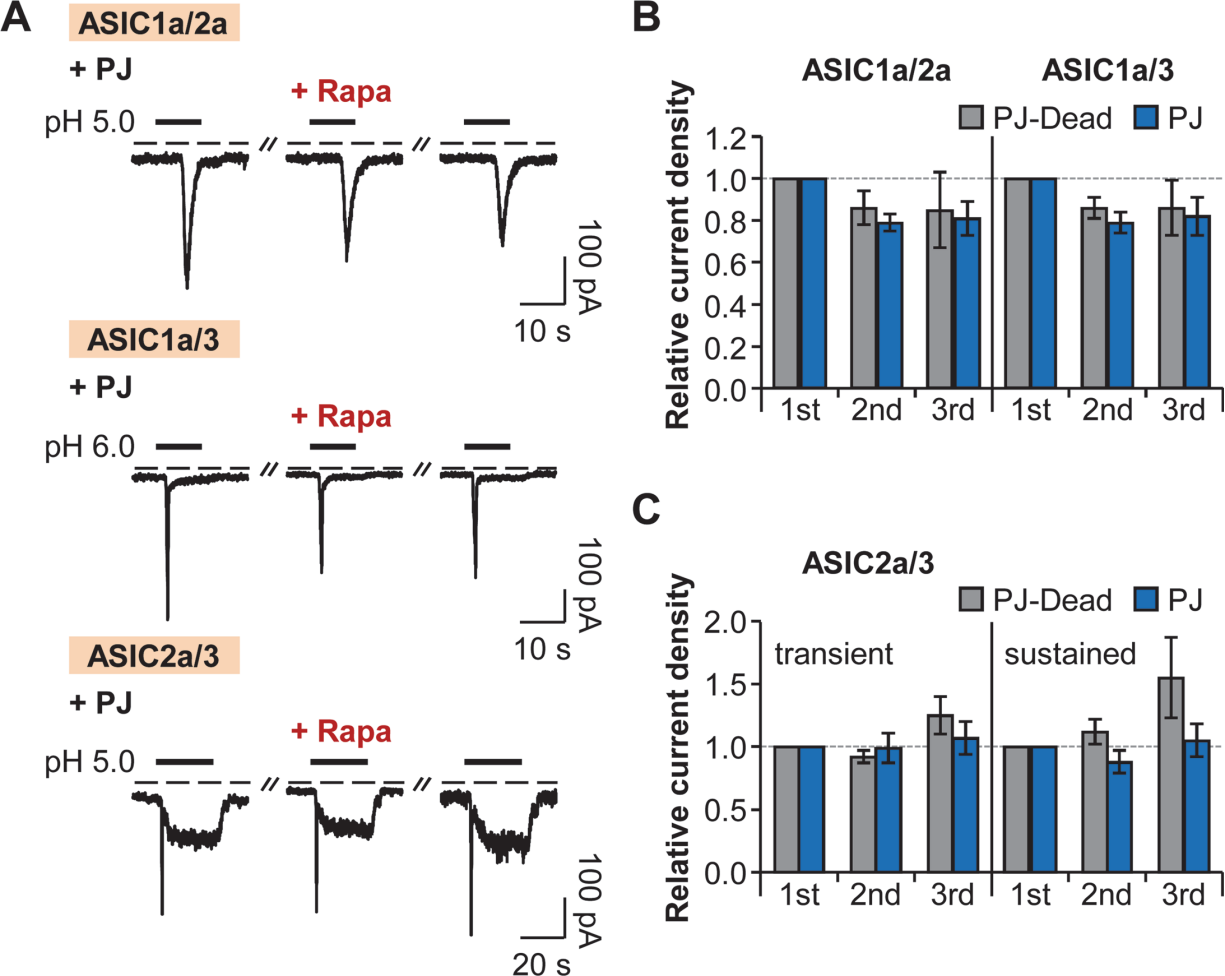
Heteromeric ASIC currents are insensitive to PI(4)P and PI(4,5)P_2_. (A) Current traces from ASIC1a/2a, ASIC1a/3, and ASIC2a/3 heteromeric channels evoked by extracellular acidification in cells expressing LDR and PJ. Rapamycin (1 μM) was bath-applied for 60 s, and then normal extracellular solution was perfused for 10 s right before the second pulse to minimize possible side effects of rapamycin. Dashed line indicates the zero current level. (B) Relative current density measured for the currents of ASIC1a/2a and ASIC1a/3 in (A) (n = 3, respectively). Current density of each pulse was divided by that of the first pulse. There is no statistical significance with two-way ANOVA followed by Bonferroni post-hoc test. (C) Relative current density measured for the transient and sustained currents of ASIC2a/3 in (A) (n = 3 for PJ-Dead; n = 5 for PJ). There is no statistical significance with two-way ANOVA followed by Bonferroni post-hoc test. Data are mean ± SEM.

We also tested whether heteromeric ASICs have dependence on phosphoinositides for their function, since most ASICs exist as heteromeric channels in physiological conditions [42–44]. The current traces from heteromeric channels of ASIC1a/2a, ASIC1a/3, and ASIC2a/3 were similar to those of a previous study [45]. Recruitment of PJ to the PM had no significant effects on either ASIC1a/2a or ASIC1a/3 heteromeric channels (Fig. 3A-B), and transient and sustained currents of ASIC2a/3 heteromeric channels were not significantly affected by the application of rapamycin (Fig. 3A and C). In conclusion, neither homomeric ASICs nor heteromeric ASICs require phosphoinositides for their activities.

### Neither ASICs nor TRPV1 Activities are Affected by Depletion of PI(3,4,5)P_3_

**Fig 4:**
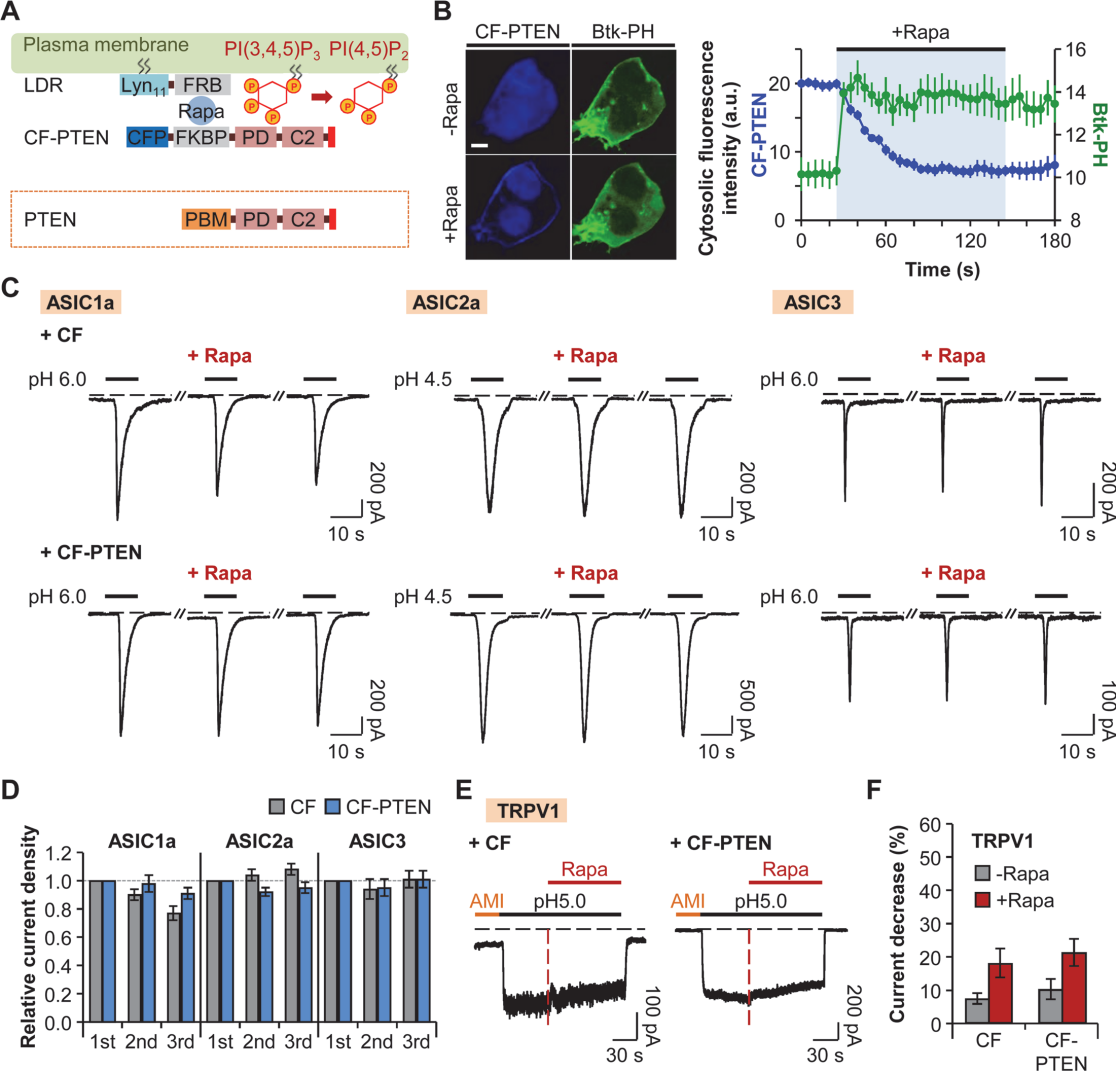
Neither ASICs nor TRPV1 activities are affected by depletion of PI(3,4,5)P_3_. (A) CF-PTEN is rapidly recruited to the plasma membrane anchor LDR by dimerization of FRB and FKBP upon addition of rapamycin, and the PD domain of CF-PTEN specifically dephosphorylates PI(3,4,5)P_3_ to PI(4,5)P_2_. Red bar in the C-terminal tail of PTEN indicates PDZ-binding domain. (B) Confocal images of cells expressing LDR, CF-PTEN, and Btk-PH-GFP before and after the addition of rapamycin (1 μM) for 120 s (Scale bar, 5 μm) and cytosolic fluorescence intensities of CFP (*blue*) and GFP (*green*) (n = 3). The values of the Y-axis use an arbitrary unit. (C) ASIC current traces triggered by extracellular acidification in cells expressing LDR and CF (lacking PTEN) or CF-PTEN. Rapamycin (1 μM) was bath-applied for 60 s, and then normal extracellular solution was perfused for 10 s right before the second pulse to minimize possible side effects of rapamycin. Dashed line indicates the zero current level. (D) Relative current density measured for the cells in (C) (CF (n = 8), CF-PTEN (n = 7) for ASIC1a; CF (n = 8), CF-PTEN (n = 8) for ASIC2a; and CF (n = 10), CF-PTEN (n = 10) for ASIC3). Current density of each pulse was divided by that of the first pulse. There is no statistical significance with two-way ANOVA followed by Bonferroni post-hoc test. (E) TRPV1 currents in response to pH drop in the cells expressing LDR and CF or CF-PTEN. Rapamycin (1 μM) was co-applied for 90 s during the acid stimuli. Amiloride (300 μM) was pretreated for 30 s before the pH pulse. Black dashed line indicates the zero current level. Red dashed line indicates the point of rapamycin application. (F) Percentage of current decrease in (E) during 45 s of acidification before (grey) and after (red) rapamycin addition (n = 9 for CF; n = 10 for CF-PTEN). Data are mean ± SEM.

Even though the PJ system is a powerful tool for probing the role of phosphoinositides for the function of ion channels, it has a limitation in terms of investigating the specific effect of PI(3,4,5)P_3_ on the channels. Therefore, we generated a novel chimeric protein to further investigate the role of PI(3,4,5)P_3_ in the activities of proton-sensitive ion channels. One of the tumor suppressor genes, PTEN (phosphatase and tensin homologue deleted on chromosome 10) codes a cytosolic 3-phosphatase that degrades PI(3,4,5)P_3_ by removing the phosphate at the D3 position of the inositol ring [46–48]. PTEN has a substrate specificity toward PI(3,4,5)P_3_ [49] and is composed of N-terminal phosphoinositide-binding motif (PBM) that contributes to the recruitment of PTEN to the PM, phosphatase domain (PD), C2 domain (C2) that binds to PM phosphatidylserine (PS), and C-terminal tail PDZ-binding domain [36,46–48] (Fig. 4A). We inserted the region from PD to C-terminal tail of PTEN to the C-terminus of CFP-FKBP (CF) (Fig. 4A). CFP-FKBP-PTEN (CF-PTEN) was translocated to the PM anchor LDR upon addition of 1 μM rapamycin; in turn, PI(3,4,5)P_3_ was depleted as shown by the specific PI(3,4,5)P_3_ probe, PH domain of Bruton tyrosine kinase (Btk) (Btk-PH-GFP) (Fig. 4B). The cytosolic fluorescence intensity of Btk-PH rapidly increased while that of CF-PTEN gradually declined by rapamycin addition (Fig. 4B), indicating the successful development of a novel translocatable 3-phosphatase tool.

Using this tool, we tested the sensitivities of homomeric ASICs and TRPV1 channels to PM PI(3,4,5)P_3_. Although the membrane PI(3,4,5)P_3_ was depleted following the addition of rapamycin, the repetitively activated currents of homomeric ASIC1a, ASIC2a, and ASIC3 channels remained unchanged (Fig. 4C-D). These results suggest that ASICs are insensitive to the depletion of PI(3,4,5)P_3_ in intact cells. Next, we investigated whether the depletion of membrane PI(3,4,5)P_3_ in an intact cell affects the proton-activated TRPV1 currents. The current decrease during 45 s of acidification right before the addition of rapamycin was not significantly different between two groups (CF: 8 ± 2% (n = 9) and CF-PTEN: 10 ± 3% (n = 10)) (Fig. 4E-F). In the presence of rapamycin, TRPV1 currents were decreased by 18 ± 4% (n = 9) or 21 ± 4% (n = 10) in cells expressing CF or CF-PTEN, respectively, during 45 s of acidification (Fig. 4E-F). Difference in current decrease ratio between the two groups was not statistically significant. These results indicate that the depletion of PI(3,4,5)P_3_ has no effects on the activities of TRPV1 channels, although TRPV1 currents are inhibited by simultaneous depletion of both PI(4)P and PI(4,5)P_2_. This result is supported by the previous reports suggesting that PI(3,4,5)P_3_ is not an endogenous regulatory factor of TRPV1 currents [27,28]. In inside-out patches, the application of pH domain of the general receptor of phosphoinositides 1 (GRP1) which selectively binds to PI(3,4,5)P_3_ did not inhibit the TRPV1 currents, while the application of PLCδ1-PH produced the decrease of the currents [27]. Moreover, in a reconstituted liposome, PI(3,4,5)P_3_ did not have any effect on the TRPV1 currents, unlike other phosphoinositides [28]. Taken together, neither ASICs nor TRPV1 activities are altered by depletion of PI(3,4,5)P_3_.

### ASICs and TRPV1 Channels are Differentially Regulated by AA

In the data above, we investigated the regulatory effects of PM phosphoinositides on proton-sensitive ion channels, ASICs and TRPV1, and discovered a difference in their sensitivities toward phosphoinositides. We next asked how these proton-sensitive ion channels are regulated by arachidonic acid (AA), which is liberated from the membrane phospholipids by phospholipase A_2_ (PLA_2_) activity [50,51]. A pro-inflammatory mediator, AA is a polyunsaturated fatty acid acting as a lipid second messenger [1,52]. Several ion channels, including ASICs [53,54] and TRP channels [28,55], are known to be regulated by AA either by a direct or indirect action [52]. Here, we investigated whether AA also differentially regulates the activities of ASICs and TRPV1 channels as do phosphoinositides.

**Fig 5:**
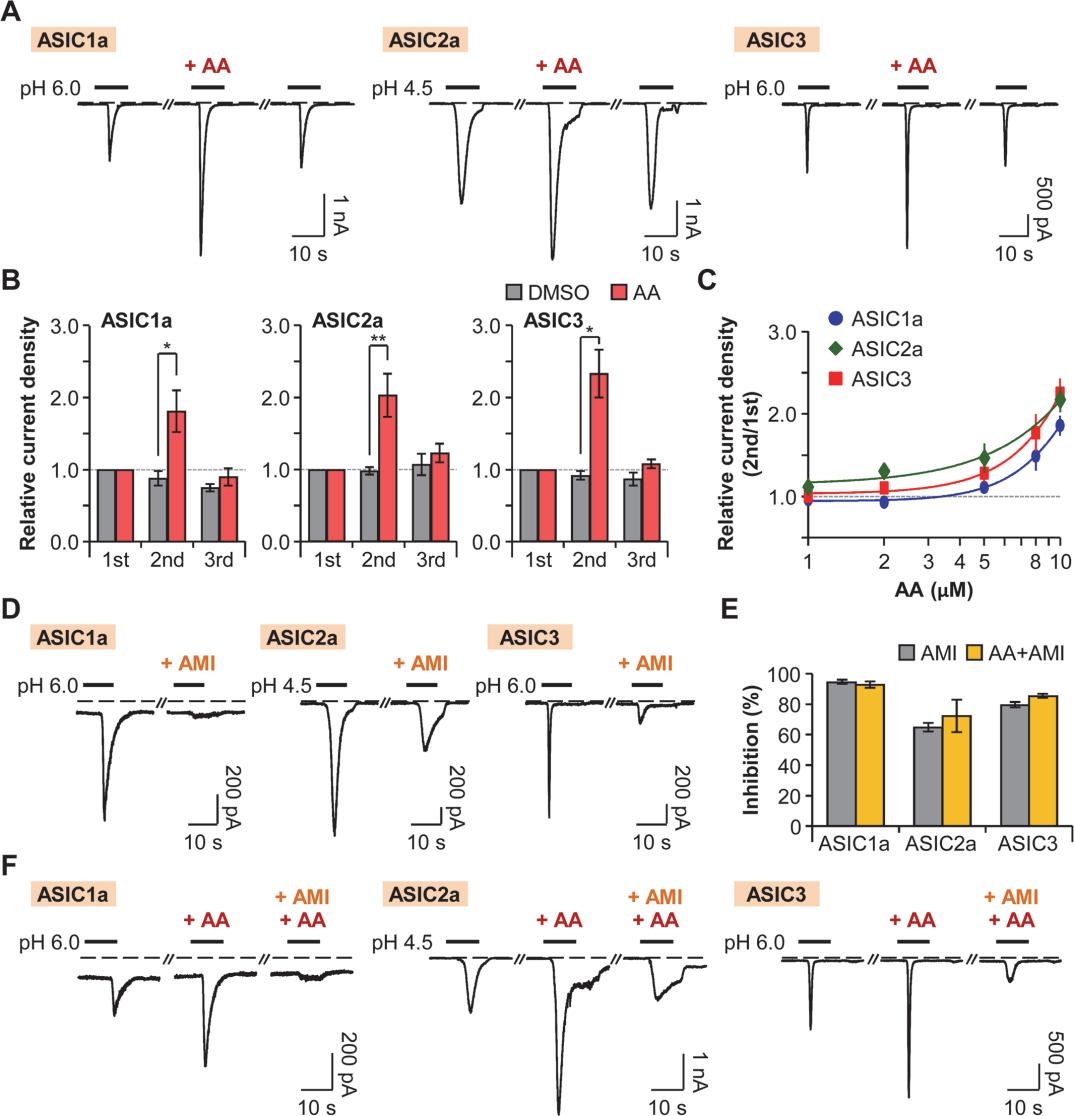
Potentiation of ASICs by AA. (A) ASIC current traces activated by rapid extracellular pH changes. AA (10 μM) was bath-applied for 20 s before the second pulse. Dashed line indicates the zero current level. (B) Relative current density was measured for the cells expressing ASIC1a (n = 5 for DMSO; n = 6 for AA), ASIC2a (n = 5 for DMSO; n = 10 for AA), and ASIC3 (n = 5 for DMSO; n = 12 for AA). Current density of each pulse was divided by that of the first pulse. * *P* < 0.05 and ** *P* < 0.01, with two-way ANOVA followed by Bonferroni post-hoc test and student’s *t*-test. (C) Dose-dependent relative current density of ASIC1a (*blue*) (n = 5–20), ASIC2a (*green*) (n = 5–25), and ASIC3 (*red*) (n = 5–23). (D) ASIC1a and ASIC3 currents were inhibited by preincubation of cells with pH 7.4 solution containing amiloride (300 μM) for 20 s before the second pulse. In the case of ASIC2a, 600 μM of amiloride was applied for 30 s before and during the second pulse. (E) Percentage of inhibition by amiloride in the absence (grey) or the presence (yellow) of AA (AMI (n = 7) and AA+AMI (n = 4) for ASIC1a; AMI (n = 4) and AA+AMI (n = 3) for ASIC2a; and AMI (n = 6) and AA+AMI (n = 6) for ASIC3). (F) The potentiating effect of AA (10 μM) on ASIC currents was inhibited by amiloride. Data are mean ± SEM.

When the extracellular solution containing 10 μM of AA was perfused for 20 s right before the second pH pulse, the peak current density of the second pulse in cells transiently expressing homomeric ASIC1a channels was increased by 81 ± 29% (n = 6) compared to that of the first pulse (Fig. 5A-B). On the other hand, the peak current density of the second pulse was slightly decreased in the control group (Fig. 5B). We observed that the potentiating effect of AA is reversible, and the peak current density was recovered to the initial level after washout of AA (Fig. 5A-B). In the cells expressing ASIC2a homomeric channels, the peak current density of the second pulse was reversibly increased by 103 ± 30% (n = 10) compared to that of the first pulse, whereas, in the control group, the difference in the current density between the first and the second pulses was negligible (Fig. 5A-B). Similarly, the peak current density of the second pulse in cells expressing ASIC3 homomeric channels was reversibly increased by 133 ± 33% (n = 12) compared to that of the first pulse (Fig. 5A-B). AA increased the respective ASIC currents in a dose-dependent manner (Fig. 5C). ASIC1a, ASIC2a, and ASIC3 homomeric channels displayed similar dose-dependent curves.

Next, we also tested whether the potentiated currents by AA are attributable to other non-specific currents in tsA201 cells. First, we observed that respective ASIC currents were inhibited by amiloride. ASIC1a currents were inhibited by 95 ± 1% (n = 7) when the extracellular solution containing 300 μM of amiloride was perfused for 20 s right before the second pH pulse (Fig. 5D-E). AA (10 μM)-induced increased ASIC1a currents were also inhibited by 93 ± 2% (n = 4) by co-application of AA and amiloride before the second pH pulse (Fig. 5E-F). In the case of ASIC2a homomeric channels, the currents were inhibited by 65 ± 3% (n = 4) when amiloride (600 μM) was applied for 30 s before and during the second pH pulse (Fig. 5D-E). The potentiating effects of AA (10 μM) on ASIC2a currents were inhibited by 72 ± 11% (n = 3) by amiloride (Fig. 5E-F). ASIC3 currents were inhibited by 80 ± 2% (n = 6) by preincubation of cells with extracellular solution containing 300 μM of amiloride for 20 s before the second pH pulse (Fig. 5D-E), and the potentiating effects of AA (10 μM) were similarly inhibited by 85 ± 1% (n = 6) (Fig. 5E-F). Collectively, our results suggest that AA reversibly potentiates homomeric ASIC currents.

**Fig 6:**
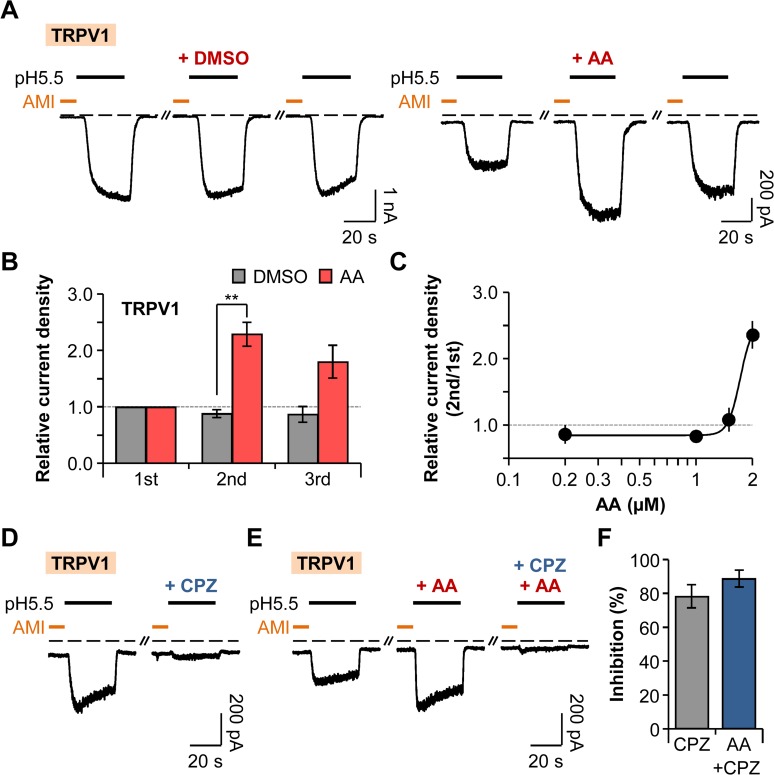
Potentiation of TRPV1 by AA. (A) TRPV1 current traces repetitively activated by extracellular pH drop to 5.5 for 30 s with time intervals of 300 s. Amiloride (300 μM) was pretreated for 10 s before the pH pulses. AA (2 μM) was applied for 20 s before the second amiloride treatment. Dashed line indicates the zero current level. (B) Relative current density was measured for the cells in (A) (n = 4 for DMSO; n = 9 for AA). Current density of each pulse was divided by that of the first pulse. ** *P* < 0.01, with two-way ANOVA followed by Bonferroni post-hoc test and student’s *t*-test. (C) Dose-dependent relative current density of TRPV1 (n = 5–17). (D) TRPV1 currents were inhibited by preincubation of cells with pH 7.4 solution containing capsazepine (10 μM) for 20 s before the second amiloride treatment. (E) The potentiating effect of AA (2 μM) on TRPV1 currents was inhibited by capsazepine. (F) Percentage of inhibition by capsazepine in the absence (grey) or the presence (blue) of AA (n = 6 for CPZ; n = 6 for AA+CPZ). Data are mean ± SEM.

At this time, we investigated the effect of AA on proton-activated TRPV1 currents. The bath-application of AA (2 μM) for 20 s right before the second addition of amiloride increased the current density of the second pulse by 129 ± 21% (n = 9) compared to that of the first pulse, whereas the difference in the current density between the first and the second pulses were insignificant (Fig. 6A-B). Unlike ASIC currents, AA-induced potentiated TRPV1 currents were not fully recovered to the initial level (Fig. 6A-B). AA potentiated the TRPV1 currents in a dose-dependent manner (Fig. 6C). We also tested whether these potentiated currents are indeed attributable to TRPV1 channels. TRPV1 currents were inhibited by 78 ± 7% (n = 4) by bath-application of capsazepine (CPZ) (10 μM), a specific TRPV1 inhibitor for 20 s (Fig. 6D and F). Co-application of capsazepine and AA (2 μM) before the second amiloride treatment inhibited the potentiating effect of AA on TRPV1 currents by 89 ± 5% (n = 4) (Fig. 6E-F). Taken together, AA enhances the activities of TRPV1 channels, although the recovery aspect differs from that of ASICs (Figs. 5B and 6B).

## Discussion

In this study, we observed that the activities of ASICs are independent of membrane phosphoinositides such as PI(4)P, PI(4,5)P_2_, and possibly PI(3,4,5)P_3_ by using the translocatable phosphatase system. This is the first report to show the sensitivities of ASICs toward phosphoinositides with direct manipulation of membrane phosphoinositides in intact cells. On the other hand, studies about TRPV1 sensitivity toward phosphoinositides have been undertaken by various research groups. In our system, we observed that proton-activated TRPV1 currents can be regulated by simultaneous manipulation of PI(4)P and PI(4,5)P_2_ level, not PI(3,4,5)P_3_ level, in intact cells.

Although both ASICs and TRPV1 channels can sense proton-mediated signaling, their expression and biophysical properties are different. ASICs are widely distributed throughout the CNS and the PNS, and they act as transducers of multiple sensory and synaptic signaling. TRPV1 is highly expressed in peripheral nerve endings of primary sensory neurons. Multimodal response of TRPV1 to various noxious stimuli elicits pain and leads to the development of hyperalgesia [13]. According to previous studies, ASICs and TRPV1 are co-localized in a great part of subpopulation of DRG neurons, although there are differences between species [56,57]. In rat DRG neurons, ASIC1a and ASIC3 transcripts were detected in approximately 40–45% and 30% of the TRPV1-positive neurons, respectively [56]. In these subsets of native sensory neurons, it is highly possible that the currents elicited by tissue acidosis are contributed from both ASICs and TRPV1 channels.

It is noteworthy that two channels are activated by different ranges of pH value. TRPV1 is activated by more severe acidification (pH_0.5_ activation of 5.4) than that required to activate most ASIC subunits [1,58]. ASIC1a and ASIC3 are sensitive to moderate pH drop (pH_0.5_ activation of 6.2–6.8 for ASIC1a and 6.2–6.7 for ASIC3), whereas ASIC2a requires more severe acidification for activation (pH_0.5_ activation of 3.8–5.0) [1]. Interestingly, extracellular protons have a dual effect on TRPV1 currents. At low pH (< 6.0), protons themselves activate the channel at room temperature. However, protons potentiate the channel already opened by other stimuli (capsaicin or heat) by lowering the threshold for channel activation at higher pH levels (6–7.4) [7,9,58]. Therefore, ASICs are considered main mediators of pain induced by moderate tissue acidosis, while TRPV1 is thought to contribute to more severe acidosis-mediated pain perception, together with ASICs in peripheral sensory neurons [3]. Hence, the complementary roles of these two proton-sensitive ion channels, ASICs and TRPV1, have great significance for perception of pH changes; thus, to understand the regulatory mechanisms of those channels is quite important.

Phosphoinositides have emerged as general regulators of ion channels, and PI(4,5)P_2_ is known to stabilize the open state of many ion channels [1,14,17–23]. However, regulation of TRPV1 by phosphoinositides is controversial [10,11,24–30]. In this study, we simply tested the effects of phosphoinositides on proton-activated TRPV1 currents by using a rapamycin-inducible PJ system, and compared the results with that of ASICs. We observed that proton-activated TRPV1 currents are significantly inhibited by the recruitment of PJ, while the translocation of INPP5E had no statistically significant effect on the currents (Fig. 1). These results are consistent with the study by Hammond et al. (2012) [35]. They observed that capsaicin-activated TRPV1 currents in HEK293 cells are inhibited when both PI(4)P and PI(4,5)P_2_ are depleted by the translocation of PJ [35]. In contrast to TRPV1 channels, the function of ASICs does not rely on PM phosphoinositides. By using the PJ system, we confirmed that homomeric ASIC1a, ASIC2a, and ASIC3 channels and heteromeric ASIC1a/2a, ASIC1a/3, and ASIC2a/3 channels are insensitive to PI(4)P and PI(4,5)P_2_. These results are, in fact, unexpected since one previous study reported that activation of M_1_R by its agonist, oxotremorine-M (Oxo-M), inhibited ASIC currents in Chinese hamster ovary (CHO) cells heterologously expressing ASIC1a and M_1_R, and also in isolated rat hippocampus CA1 and striatum interneurons [31]. That study suggested that muscarinic inhibition of ASIC1a currents could be due to depletion of PI(4,5)P_2_ available to the channel [31]; however, we observed no dependence of ASICs on PI(4)P or PI(4,5)P_2_ (Figs. 2 and 3). Therefore, it is possible that inhibition of ASIC currents by M_1_R activation might occur through mechanisms other than direct action of PI(4,5)P_2_ hydrolysis. We also tried to test whether the activation of M_1_R by Oxo-M modulates the function of homomeric ASIC1a channels in either tsA201 or CHO cells. However, we observed no inhibition of ASIC1a currents by M_1_R activation in either type of cells (unpublished observations). In another previous study by Li et al. (2012), they found that supplementing the pipette solution with a short-chain PIP_2_ was not effective to decrease the desensitization of ASIC1a currents. Furthermore, they observed that the currents were not regulated by activation of muscarinic receptor [34]. The discrepancy between the studies is not clear; however, in our system, using the translocatable PJ system, we verified that the activities of ASICs are independent from PM phosphoinositides.

We also investigated the dependence of ASICs and TRPV1 channels on PI(3,4,5)P_3_. ENaC and some families of TRP channels such as TRPM4 are known to have sensitivities toward PI(3,4,5)P_3_ as well as PI(4,5)P_2_ [20–22,59]. To selectively dephosphorylate PI(3,4,5)P_3_, we generated a novel engineered phosphatase tool from PTEN, which is a well characterized 3-phosphatase that prefers PI(3,4,5)P_3_ as a substrate [49]. By using a chimeric protein CF-PTEN, we observed that neither ASICs nor TRPV1 currents were altered by depletion of membrane PI(3,4,5)P_3_ in intact cells.

Finally, we compared differential regulatory features of ASICs and TRPV1 by AA, a pro-inflammatory mediator released from phospholipids. In our experiments, AA induced significant potentiation of all ASIC1a, ASIC2a, ASIC3, and TRPV1 channels. TRPV1 currents were particularly more sensitive to AA than ASIC currents were. It is noteworthy that potentiated ASIC currents by AA were, remarkably, almost fully recovered to the initial level of the currents, while TRPV1 currents were partially recovered after washout of AA (Figs. 5 and 6). We propose that this is likely due to the difference in regulatory mechanisms of those channels by AA. The potentiating effect of AA on TRPV1 currents has been reported to result from the AA metabolites of lipoxygenase pathways, such as 12- and 15-hydroperoxyeicosatetraenoic acid (HPETE) [60,61]. However, a recent study suggested that both AA and its metabolites can produce marked sensitization of TRPV1 currents in the reconstituted liposome, allowing them to observe the direct effects of factors in the absence of other cellular enzymes [28]. Therefore, AA seems to regulate the TRPV1 channels by both a direct and an indirect action through the metabolism pathways. On the other hand, ASICs are thought to be directly regulated by AA [54]. One previous study reported that inhibition of lipoxygenase or cyclooxygenase pathway did not impair the potentiating effect of AA on ASIC currents [54]. Therefore, ASICs and TRPV1 channels are differentially regulated by a pro-inflammatory mediator AA.

In this study, we observed that two proton-sensitive ion channels, ASICs and TRPV1, display differential regulatory features by membrane phosphoinositides and AA. Their different topology, distribution, and biophysical properties might establish different sensitivities toward phospholipids. Understanding the relationship between these two groups of channels, and their relative contributions in proton-mediated signaling, is quite important for comprehending the complementary roles of ASICs and TRPV1 in the nervous system.
